# Monthly Summary

**Published:** 1871-09

**Authors:** 


### MONTHLY SUMMARY.

---

*To Crystallize Plants, Flowers, &c.*—Dissolve 18 oz. of pure alum in a quart of distilled water by the aid of a boiling heat. When the solution is nearly cool, suspend in it, by a thread of silk, or fine twine, the objects to be crystallized for about twenty-four hours; they are then to be taken out and suspended in a dry, shady room till thoroughly dry. The proper temperatute for the solution in which the object is to immersed is about 95 degress, F. When the solution is too cold, the crystals formed will be too large; and when too hot they will be too small. If different colors are desired, they may be given by boiling in the alum solution a little indigo, logwood, French berries, or a soluble vegetable or mineral dye, whose chemical nature does not decompose or is not decomposed by the alum. Among the subjects adapted to this purpose, may be mentioned the moss rose, ears of wheat, barley, oats and other cereals; the grasses, and any variety of flowers; various insects, such as are interesting in the study of etymology; and above all, the most endless variety of sea-weeds and mosses, which form one of the most beautiful features in the study of botany. For parlor ornaments, especially in the country, these preparations are vastly superior to, and more attractive than the more costly works of art.—*Jour. App. Chem.*

---

*Effects of Alcohol and Tobacco on the Sight.*—On the subject of color-blindness and amblyopia, Dr. Richard H. Derby (*N. Y. Medical Journal*) says: "Almost always both eyes are affected. This form of amblyopia occurs almost solely in men: out of fifty-six cases only three were women. It is a disease of adults; its frequency increasing from the twentieth to the fortieth year. In a portion of the cases abuse of alcohol was certainly the cause of the affection, and in others the excessive use of tobacco undoubtedly contributed to produce the disease. Forster, in a paper on the injurious action of tobacco on the vision, attaches still greater importance to this agent, as a cause of amblyopia, supporting the views of Mackensie Sichel, Hutchinson, Lureiro, and others. The author cites twenty cases, in which there was a central scotoma, with a horizontal diameter of 18° to 25°, within which large letters could still be recognized. All of these patients suffered from some affection of the digestive and nervous system. Loss of appetite, constipation, loss of sleep, were common symptoms. Each one of the twenty patients was a strong smoker, and in eleven of these cases a very marked improvement was observed when the use of tobacco was given up."

---

*Poisoning by Hydrate of Chloral—Strychnia as an Antidote.*—Dr. Charles Marshall reports a child, one year old, had been suffering from general irritability, complicating a prolonged diarrhœa. At 12 o'clock a mixture containing three grains of chloral was ordered, one-third of the whole mixture to be given every three hours. By a mistake of the nurse the whole three grains were given at once. At 8 o'clock the patient was again seen. The pupils were contracted, respiration gasping, with general prostration. 1-32 grains of strychnia was now administered as an antidote, but no good result followed. To ward off the approaching syncope, a cold plunge bath was given, followed by a mustard bath, but without avail.

Child died at ten o'clock, ten hours after taking the fatal dose. At the post-mortem the mucous membrane of the stomach was found normal, but in it there was a marked odor of the hydrate.

The intestines showed intero-colitis, but it is not probable from the progress of the case that this was the determining cause of death.—*Med. and Surg Rep.*

---

*Lead-Line on the Gums.*—Dr. William Frank Smith, in the *Lancet*, writes: When I commenced practice in Sheffield, I employed the iodide of potassium as an eliminative, and while employing it made a somewhat remarkable observation, which has been since confirmed by Dr. Hilton Fagge. A man came under my care with severe lead palsy. On examining his mouth, I was surprised, to find the lead line absent; there was no trace of it, though I looked carefully with a good lens. I pointed this out to the then house surgeon, Mr. Cooper, and to one of my colleagues. The patient was placed under the iodine treatment —doses, ten grains three times a day. In the course of a week or two I was very much surprised to see little arches of black puncta over the roots of the teeth, and in the course of a few more days the blue line was well marked, disappearing again before he left the hospital, about six weeks after his admission.

---

*The Formation of Pus.*—Dr. Hartman gives his views in regard to the formation of pus. The pus cell he believes to be identical with the white blood cell and with the lymph cell, and, like these, to be formed from the elements of the lymph and chyle. "I believe," he says, "that pus cells are only white blood or lymph cells, but that they do not come out of the circulation, but are developed where they are found, at the seat of the inflammation." Inflammation gives rise to an arrest of the circulation in the beginninig of the lymphatics, in the vasa serosa, in the connective tissue cells, and even in the capillaries. This it is which gives rise to the inflammatory swelling. A development of lymph cells (pus cells) now takes place in the stagnating lymph contained in the lymphatics, and also in the connective tissue cells; finally the latter burst, and the fully-formed and immature pus cells are extravasated. This is the process of the formation of pus as it is observed in the connective tissue cells.

---

*No Life without Phosphorus.*—Dr. Frandlin has been making some experiments upon the developement of fungus growth in potable water; and, as a result of his labors, arrives at the following conclusions:

"1. Potable waters mixed with sewage, urine, albumen and certain other matters, or brought into contact with animal charcoal, subsequently develop fungoid growths and other organisms, when small quantities of sugar are dissolved in them and they are exposed to a summer temperature.

"2. The germs of these organisms are present in the atmosphere, and every water contains them after momentary contact with the air.

"3. The development of these germs cannot take place without the presence of phosphorus in some form of combination. Water, however, much contaminated, if free from phosphorus, does not produce them. A German philosopher has said, 'without phosphorus, no thought,' which may now be changed to without phosphorus, no life.' "—*Med. and Surg. Reporter.*

---

*A New Styptic.*—Collodion. 100 parts; carbolic acid, 10 parts; tannin (Pelouse's), 5 parts; benzoic acid (from gum), 5 parts.

Mix the ingredients in the order above written, and agitate until perfect solution is effected. This preparation has a brown color, and leaves on evaporation a strongly adherent pellicle. It instantly coagulates blood, forming a consistent clot, and a wound rapidly cicatrizes under its protection.—*Carlo Parvesi.*

